# Homologies between Bauhinia forficata Link subsp. pruinosa and pancreatic beta-cell specific transcriptional activator: a starting point for drug design new in diabetes?

**DOI:** 10.1186/1758-5996-7-S1-A245

**Published:** 2015-11-11

**Authors:** Luis Jesuino de Oliveira Andrade, Gabriela Correia Matos de Oliveira, Paulo Roberto Santana de Melo, Hudson Sá Sodré, Carlos Alberto Menezes, Alcina Maria Vinhaes Bittencourt

**Affiliations:** 1 Universidade Estadual de Santa Cruz – Bahia, Itabuna, Brazil

## Background

Diabetes mellitus (DM) is a chronic disease with an ever-increasing incidence in world and has become the object of scientific research into the search for novel therapeutic alternatives. Bauhinia forficata Link, known locally as cow foot, has been traditionally used as tea in folk medicine of Brazil for treatment of DM.

## Objective

The purpose of this study is to explore the possible homology between the AA sequences of ribulose 1,5-biphosphate carboxylase large subunit, partial (chloroplast) [Bauhinia forficata subsp. pruinosa] and pancreatic beta-cell specific transcriptional activator [Homo sapiens], using databanks of proteins of National Center for Biotechnology Information (NCBI).

## Materials and methods

Were performed the comparison between the AA sequence of the GenBank: CAA94019.1-ribulose 1,5-biphosphate carboxylase large subunit, partial (chloroplast) [Bauhinia forficata subsp. pruinosa] and GenBank: BAC20389.1-pancreatic beta-cell specific transcriptional activator [Homo sapiens], available in the database of NCBI with the Basic Local Alignment Search Tool (BLASTp) software.

## Results

**Figure 1:**
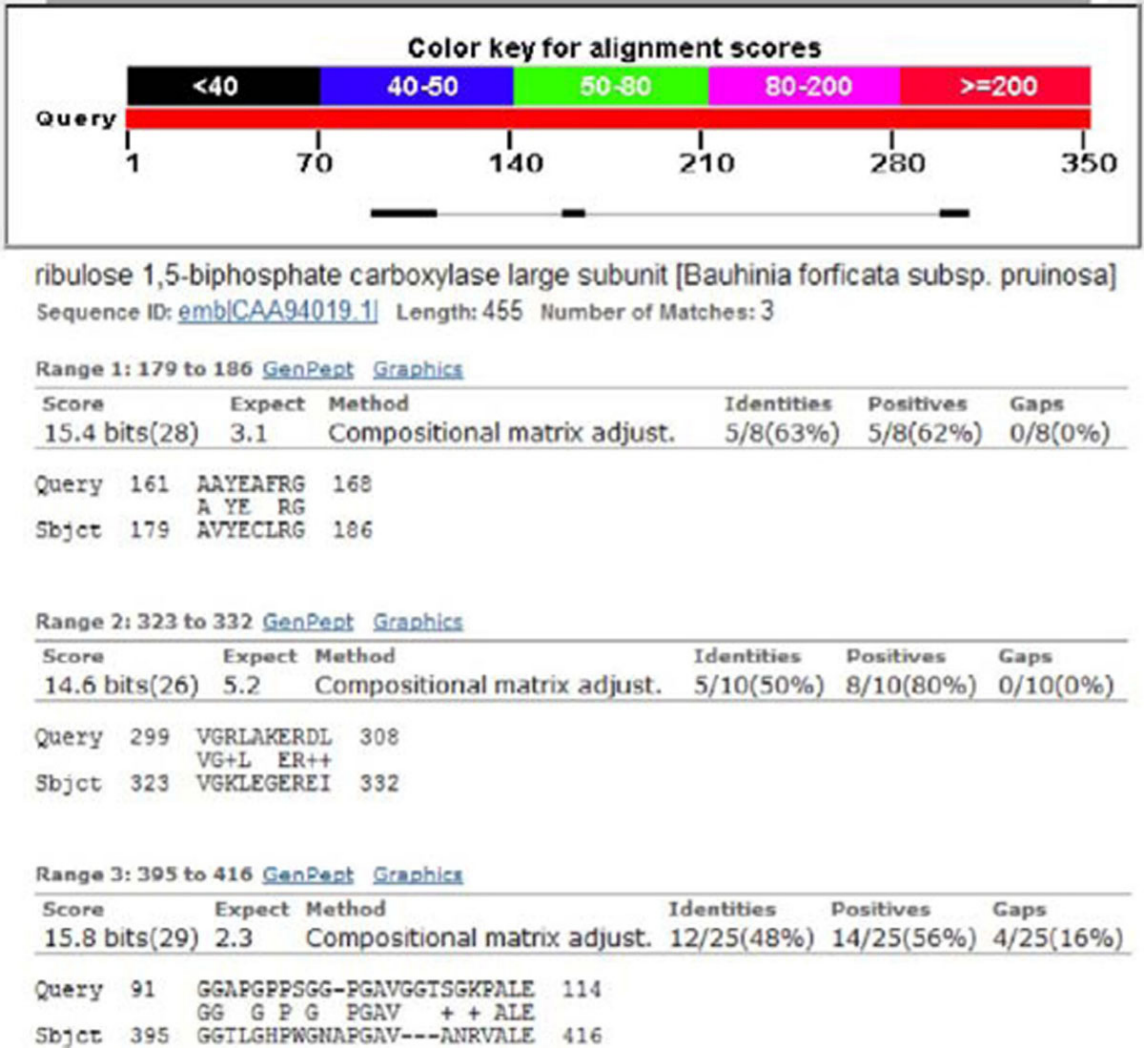
Homologies between bauhinia forficata link subsp., pruinosa and pancreatic beta-cell specific transcriptional activator.

The homology between the ribulose 1,5-biphosphate carboxylase large subunit, partial (chloroplast) [Bauhinia forficata subsp. pruinosa] and the pancreatic beta-cell specific transcriptional activator [Homo sapiens] ranged from 48.0% to 63.0% (Fig.1).

## Conclusion

Bioinformatics data, suggest a possible pathogenic link between ribulose 1,5-biphosphate carboxylase large subunit, partial (chloroplast) [Bauhinia forficata subsp. pruinosa] and pancreatic beta-cell specific transcriptional activator [Homo sapiens], and studies on this plant are important to the safe, effective development of pharmaceutical products for the treatment of diabetes.

